# Improvising livestock service in hilly regions through indigenous wisdom towards control of tick infestation: Institutional relationships

**DOI:** 10.14202/vetworld.2018.687-692

**Published:** 2018-05-24

**Authors:** Khumaji Badaji Kataviya, Bharat Parmar, Ramesh Patel, Pranab Jyoti Das, Vivek Kumar, Amit Mahajan, Ravinder Singh, Devesh Thakur, Amol Kinhekar, R. K. Ravikumar, Vipin Kumar

**Affiliations:** 1 National Innovation Foundation-India, Amarapur, Gandhinagar, Gujarat, India; 2 Society for Research and Initiatives for Sustainable Technologies and Institutions, Ahmedabad, Gujarat, India; 3 Department of Animal Husbandry, Sirmaur, Himachal Pradesh, India; 4 Department of Veterinary and Animal Husbandry Extension, Dr. GC Negi College of Veterinary and Animal Sciences, Palampur, Himachal Pradesh, India; 5 NIF Cell, Dehradun, Uttarakhand, India

**Keywords:** acaricide, indigenous, institution, livestock, ruminant, tick

## Abstract

**Aim:**

This study was conducted to demonstrate the acaricide efficacy of novel indigenous veterinary medication shared by an outstanding knowledge holder against naturally infested cattle and efforts in mainstreaming such wisdom.

**Materials and Methods:**

An indigenous herbal medication in control of tick infestation was documented, and experimentation was held against naturally affected cattle. Eighteen clinically infested cattle population comprising 16 crossbred and 2 non-descript cattle were purposively selected. Majority of them were adult females, reported with a higher incidence of tick at Veterinary institution. The average pre-treatment tick count at 24 sites of observations among these animals was 18.91±2.04 (Mean [x̄]±standard error [SE]). The medication was topically applied once daily for 2 days and post-treatment observations were recorded for an experimental period of 14 days’ duration.

**Results:**

During 24-h post-treatment observation, the medication had shown 92.95% acaricidal property with clinically irrelevant rate of tick infestation of 1.33±0.39 (x̄ ±SE) was noticed before application of subsequent (second) dosage. This practice was found significantly effective at 5% level of significance (t_0.05, 23_=9.08) illustrating faster relief to livestock. Animals were treated with herbal medication as per dosage on the second day and no reinfestation was noticed up to 14 days of experimental observation.

**Conclusion:**

The study strengthens the belief that indigenous herbal acaricide can facilitate quality livestock service at geographically distant locations. These medications can provide quicker relief, minimize tick resistance and are favorable to the environment.

## Introduction

Tick infestation is a predominant problem in hilly regions occurring throughout the year [1]. An incidence rate of 61% in large ruminants and 50% in small ruminants were reported [2]. These ectoparasites affect resource-poor farming communities worldwide [3-5]. They also act as a vector for hemoprotozoan diseases incurring an economic loss to livestock farming [6]. Nature of livestock rearing patterns such as housing and prevailing environment causes severe welfare challenge to affected animals [7]. However, social realities and aspiration of community create challenges in protecting welfare of livestock against tick infestation. Farmer has to depend on medications provided/referred by Veterinary Institutions (VIs) for control of ticks. There is a need for service institutions to plan program that relies on local wealth and embracing technology which cost least inconvenience to the environment. The criteria for developing such medications depend on ease of administration, treatment, efficacy duration, and speed to demonstrate parasiticidal action [8]. At present, most available acaricides are organophosphorus compounds that pose challenges to public health hygiene. Use of such chemical formulations resulted in the development of resistance and farmers, particularly in developing countries, were affected [9]. Apart from it, lack of knowledge, inadequate veterinary service, and a decrease in availability of medications hinder tick control measures [10]. Evidences are being generated, shared in respect to inextricable link of climate changes and these vectors as well [11]. This is more pertinent in hilly terrain, wherein intervention tends to be complex and lack priority among national planners [12]. Hence, generating suitable approach in minimizing constraints and enhancing the accessibility of viable technology at farmers’ field is paramount.

Farming communities’ to meet their challenges require effective, economical, and environment-friendly technologies. Indigenous Knowledge Research System (IKRS) that is prevalent widely can complement livestock health care [13]. They form informal institutions that conduct activities outside government norms for livelihood [14]. These knowledge systems rely on reciprocal exchange, depends on endogenous decision-making among members’ of a social group [15]. This system has the capacity to overcome constraints not only at their place of origin but also elsewhere. However, limited evidence was available in upscaling indigenous technologies, and there is a need to showcase different implementation models. Acknowledgment of utility value of traditional medicine, role of farmers, and knowledge holders can endow wider application in a cost-effective manner [16,17]. Studies reiterated the importance of technology, management practices, and suitable approach of stakeholders in disease control strategies [18]. The relevance of social structure and need for appropriate sustainable technology transfer from traditional medicine has been articulated [19]. As enunciated by *Honey Bee Network*, social capital can be leveraged by advancing social cause through trust reposed among members of social network [20]. This is pertinent as there is lack of investment both in private and public sector for the development of suitable medications in livestock sector [21]. In developing countries, this service cannot afford medicinal expenditure and unavailability of necessary drugs are common [22]. Policy strategies among African, Caribbean, and Pacific Group of States refer limited institutional framework which had hindered disease control strategies [23]. Further, farmers’, particularly smallholders, cannot meet expense for quality medications limiting livestock health [24]. The abiding influence is to seek evidence in evaluating knowledge systems and elements for improvising livestock service.

Adoption of several technological activities did not head in desired direction due to limited attention to social, economic, and inherent policy issues [25]. The significance of sustaining eco-friendly technologies in hilly regions was articulated [26]. *In situ* conservation activities have to be promoted for enabling availability of medicinal plants [27]. Agriculture innovations emerge based on several elements and appreciating them help stakeholders (internal and external) to work jointly on well-defined outline [28]. Social relations among village institutions and their locus standi can elaborate these intervention activities. Hence, multidisciplinary actions enable planners to appreciate subcontextual dimensions while augmenting IKRS. It is to be noted that VIs have been playing economic and social role in facilitating livelihood of farming communities [29]. Therefore, studies call for strengthening network among creative communities, end users, and service providers [30]. This will enhance the development of concepts, theories of implementation in protecting welfare, and productivity of livestock through IKRS. Most research in livestock system depends on technologies originated elsewhere, and there is an urgent need to improvise adoption rate of medications derived from the society. Scaling up innovations or technologies require validation within locale situation [31]. IKRS provides an opportunity to reorient livestock service in farm animal health and welfare within sociocultural settings. This research aims to demonstrate utility value of societal knowledge and to share an implementation model by engaging stakeholder to minimize inadequacies in the current situation. Such empirical evidence can sensitize policies to integrate traditional healers by recognizing their bona fide efforts.

## Materials and Methods

### Ethical approval and informed consent

The prior approval from livestock owners and institutional animal ethics committee was obtained for the use of farm animals in this study which were naturally affected by tick infestation. 

### Study area, animals and experiment

This study was conducted during May-November 2016 involving documentation of indigenous medication in Sabarkantha district of Gujarat and experimentation in Sirmaur district of Himachal Pradesh, India. This study involved meeting with indigenous healer, farming community for enumerating details about medication shared by outstanding traditional knowledge holder. The animal husbandry officials and farmers’ were networked at Paonta sahib, Majra of Sirmaur District, Himachal Pradesh. Suitable intervention site, wherein animals naturally affected with clinically relevant hard tick infestation were identified. Experimental animals were selected by clinically observing infestation pattern and involvement of farmer in undertaking experimentation [32]. Specific area of infestation site on animals was observed and examination was repeated carefully. These observed parts of animal were dewlap, ear, umbilical, udder, and rear udder. These observations of tick infestation reflected that animals were affected with hard tick. A total of 18 animals (cattle) comprising 16 crossbred and 2 non-descript cattle were involved in this study based on the rate of tick infestation. Twelve of these animals were adult, one heifer and remaining five animals were calves that constituted experimental design for testing efficacy of medication (AHP/ECTO/KBK] as per healers’ dosage. About 500 g of herbal ingredients were shade-dried. The crude water extract was prepared by boiling dry herbal ingredients in 1 L fresh water. Subsequently, this preparation was allowed to cool, filtered, and used for experimentation.

Traditional medicine was applied once daily for 2 days and observed for reduction in tick infestation. Medication was provided as “pour on” topical application at the site of tick attachment among affected animals. Pre-treatment tick counts were conducted at infestation sites (n=24) before administration of medication. Counting of ticks was carried out by veterinarian through physical examination as per a method suggested by Ravikumar *et al*. [33]. All animals were observed for any untoward effect during the study period. The study tried to understand the impact of this practice against naturally occurring tick in field conditions. The comparative efficacy was calculated as per formula, T1-T2/T1×100, where T1 the pre-treatment count and T2 the post-treatment count of tick [34].

### Statistical analysis

Parasiticidal effects were analyzed statistically using t-test of matched paired observations [35] and results were interpreted.

## Results and Discussion

### Efficacy trial

Infestation of tick was observed at 24 sites on these 18 observed affected animals (n=24) identified from different farm units. Data were coded and interpreted for understanding statistical significance of outstanding healers practice in minimizing the presence of tick. The t_0.05, 23_ calculated value was 9.08 which was found to be >t_0.05, 23_ table value of 2.069 at 5% level of significance. The experimental study confirmed significant impact of indigenous medication in control of tick among naturally infested animal population. The results reinforce earlier study [36] that refers exploitation of botanicals and conserving indigenous system remains a valid approach. The findings demonstrated effective tick control strategies can be designed through IKRS.

### Parasiticidal efficacy

Experimental animals treated with indigenous preparation were examined physically on 1^st^ day, 8^th^ day, and 14^th^ day post-treatment. The applied site did not show any reactions and skin conditions were found healthy. Detachment of ticks was observed and no reinfestation was noticed during experimental period. The pre-treatment count of hard ticks observed at infested sites over 18 animals was found to be 18.91±2.04 (mean±standard error [SE]). The medication was applied as per outstanding knowledge holder dosage and impact was assessed after 24-h duration before application of the second dosage. This is an important parameter as daily percentage impact measures speedy action of medication [37]. It was observed that post-treatment count of hard tick was 1.33±0.39 (mean±SE) after 24-h duration. The total parasite count before treatment (T1) was 454 and post-treatment count was found to be 32 (T2). The calculated comparative parasiticidal efficacy was of 92.95% efficacy in clinical conditions. The presence of tick after treatment was of clinically acceptable rate under prevalent managerial situation.

### VIs and knowledge systems

The State Animal Husbandry Department (SDAH) is the major stakeholder with emphasis on animal health service in India. The research study tried to link societal knowledge with formal VI. This is pertinent as only 5.1% of farmer households were able to access information related to livestock [38]. Veterinary professionals act as the first liaison link in addressing livestock owners’ difficulties and have interpersonnel relations in village situation. This is in concurrence with earlier findings [39] as without gender prejudice livestock owners’ depend on VIs’ for livestock health and information. This reiterates importance of institutional relations between formal (VIs) and informal (traditional knowledge holders) systems in sustaining creative knowledge. The novel knowledge shared by healer has been relied by VI due to requirement for cost-effective and alternative technology in control of tick infestation. Animal husbandry professionals through their experience and professional background played a significant role in conducting trials for the benefit of farmers’. They had expressed concern over synthetic acaricides as ectoparasite (ticks) had developed resistance. Farmers’ depend on VI’s for effective livestock health care and repose faith on SDAH. Such interface among end users and service provider echoes urgency of situation. Officials from VI provided desired inputs in terms of leveraging their social contacts, clinical evaluation of affected animals as they felt the importance of such sustainable practice in hilly regions.

Eco-friendly application of technology is an important cog for control of ectoparasites [40]. Several ethnobotanical studies had made inventory of medicinal plants, its usage, and suggested for community health care [41]. However, efforts to value-add and reinforce such technical know-how through formal institutions were limited. This had created void in bolstering capabilities of communities in scaling up agricultural innovation/practices. Recognition of traditional healers is paramount in the process to conserve and utilize plant-based natural resources. Studies reflected limited availability of medicinal plants in mountain regions due to environmental changes [42] and posed constraints for local veterinary care. During this study, herbal medication was processed at farm field as per traditional healers’ know-how. This had reduced technological gap between primary users and service providers in terms of utilization of such medications. Such alternative model, namely *in-situ* incubation model of alternative technologies involving inherent knowledge of society has been shared for development of socially desirable products [43]. These are adaptive strategies to protect vulnerable communities in hilly regions by emphasizing their capacity, reconciling local wisdom.

Such comprehensive model of implementation of novel knowledge system derived from society’s knowledge ensures respect for indigenous wisdom, emotional security for farmers in protecting livestock. Emotive constraints faced by farmers due to inflamed infested site among livestock were indicated [44]. Opportunities were held in integration of local communities through knowledge; imbibe humane values of IKRS and leveraging public institutions in enhancing quality service. The theory of comprehensive technical assistance by trained resource personnel from formal system and nature of working arrangement was demonstrated. This study also illustrated ability of institutions to pass scientific knowledge in desirable format to farmers in need. Further, the paper provided a reference point in substantiating development of theories, namely non-linear innovation system, wherein technological origin featured from outside formal scientific structure.

### IKRS and livestock welfare

People in village situation depend on various stakeholders to overcome welfare issues faced by their livestock. The study illustrated the existence of knowledge system in different regions and imminent response by stakeholders to use, derive benefit. There is a need to conduct field studies to know changes in the agricultural system which influence tick beyond environmental impact [45]. Tick infestation was seldom taken into account while extending service as these welfare challenges do not cause the dramatic situation. Resource-poor livestock farmers’ may not able to offer conventional treatment cost and try to depend on indigenous knowledge [46]. Veterinary professionals also face hardships due to reoccurrence and quick development of resistance against modern medications. The situation is left with a limited choice among service provider and service seeker. Farmers’ behavior and practical measures of management have to be visualized to bring desirable impact on livestock welfare to control of tick incidence [47]. Documentation of such traditional medications was known [48]; however, this specific study enriches knowledge on reinforcement, challenges to expand, and understanding of context. In these circumstances, IKRS provides an opportunity to lessen the use of synthetic acaricides and are available in the close vicinity. The study also found that VI during experimentation had enhanced observability of this technology among potential adopters and increased rate of adoption through demonstration. These features substantiate the role of VI for being a change agent as referred by Rogers [49]. Changing strategies have to be logical step forward for empowering people and for effective livestock service system. This research had acknowledged knowledge holder and utilized their wisdom cross-regionally in control of ectoparasites.

## Conclusion

Recognizing traditional livestock herbal healers by formal sector enable quicker integration of knowledge system into practice. These medications can play a pivotal role in long-term tick control measures and are eco-friendly, an essential feature for promoting technologies in hilly regions. This study shared subcontextual characteristics of informal knowledge system and relevance of interface with formal institutions. Dovetailing technologies in farmer’s field involve relating them to veterinary professionals work experience and ability of technologies itself to convince working personnel’s. This research had exemplified a working model, wherein traditional medicine can be part of *in situ* incubation model of alternative technology. IKRS can be a transformative tool to ease reforms in livestock service. These practices lend support to veterinary professionals in incentivizing them while sharing knowledge with farmers. Effectiveness, relevance in local settings, and economic viability can enhance the scope of diffusion of this technical know-how from the perspective of livestock service institutions.

## Authors’ Contributions

KBK holds the vital knowledge, sustained and shared novel wisdom, on which this research study was conducted. RKR, VKR, AM, DT, VK participated in the discussion, drafting, and revision of the manuscript. RKR, RP, BP, PJD, AM, AK, and RS had carried out investigation(s) and experimentation. RKR and DT had leveraged institutional interface, collected literature for drafting the manuscript. RKR, VKR, DT, VK corrected errors, internalized, and designed content of the manuscript. All authors read and approved the final manuscript.
